# Top-down instruction outweighs emotional salience: nocturnal sleep physiology indicates selective memory consolidation

**DOI:** 10.3389/fnbeh.2025.1643449

**Published:** 2025-10-07

**Authors:** Laura B. F. Kurdziel, Carie Fiedler, Alex Gajewski, Caroline Pongratz

**Affiliations:** Laboratory of Social Cognition and Affective Neuroscience, Department of Psychology, Merrimack College, North Andover, MA, United States

**Keywords:** sleep, memory, directed forgetting, emotion, sleep spindles

## Abstract

**Introduction:**

Sleep plays a crucial role in memory consolidation, not only stabilizing newly encoded information but also potentially supporting forgetting. Yet it remains unclear how sleep prioritizes what is retained or discarded when multiple salience cues, such as emotional valence and top-down instructional goals, compete for consolidation.

**Methods:**

In two studies, we examined how emotional content and intentional memory instruction interact to shape memory performance across a 12 h interval that included either nocturnal sleep or wakefulness. Participants completed a directed forgetting paradigm with neutral and negatively valenced words, followed by immediate recognition and delayed free recall.

**Results:**

In both Study 1 (online) and Study 2 (in-lab), behavioral results showed that instruction to remember significantly enhanced recognition and recall, whereas emotion alone did not produce consistent benefits; however, sleep condition did not impact memory performance. In Study 2 (in-lab), which included overnight EEG monitoring, physiological markers of sleep revealed meaningful correlates of memory performance. Specifically, sleep spindle activity predicted recall for negative remember-cued words, while Slow Wave Sleep (SWS) and delta power were negatively correlated with total recall, suggesting a trade-off between deep sleep and memory accessibility. REM theta power was associated with increased false recall of emotionally negative foils, consistent with emotional memory generalization.

**Discussion:**

Importantly, these findings extend prior nap-based research by demonstrating that full-night sleep physiology reflects selective consolidation mechanisms even in the absence of overt behavioral effects. Overall, results underscore the primacy of top-down instruction over emotional salience in shaping memory, and highlight the utility of sleep physiology for understanding selective memory consolidation.

## 1 Introduction

Sleep has consistently been shown to support memory consolidation (Jenkins and Dallenbach, 1924; MacDonald and Cote, 2021; Paller and Voss, 2004; Paller et al., 2021; Rudoy et al., 2009; Stickgold, 2005). However, the ability to forget may be just as crucial to the efficiency of a healthy memory system as the ability to retain information (Anderson and Hulbert, 2021; Ryan and Frankland, 2022). In fact, several theoretical accounts have proposed that forgetting may be a primary function of sleep, helping to remove irrelevant or outdated information and reduce memory interference (Joensen et al., 2022; Lerner et al., 2021; Poe, 2017; Walker and Russo, 2004).

Understanding how sleep prioritizes what is retained and what is discarded requires examining the cues that guide memory selection. Two of the most prominent drivers of this selectivity are emotional salience and top-down cognitive instruction. Emotionally charged events, particularly those that are negative or arousing, are more likely to be remembered than neutral ones (Kensinger and Schacter, 2008; Kensinger, 2004; Adelman and Estes, 2013; Pereira et al., 2023; Kensinger, 2009), a bias believed to reflect the adaptive value of remembering threats and rewards (Mather and Sutherland, 2011; Fiacconi et al., 2015). This enhancement is often attributed to bottom-up, automatic processes involving arousal-related neuromodulatory activity, such as amygdala–hippocampal interactions (McGaugh, 2004; Mather and Sutherland, 2011). In contrast, top-down modulation reflects deliberate cognitive control over memory encoding and retrieval, as in directed forgetting, where cues to forget lead to reduced recall of specific information (Anderson and Green, 2001; Bjork, 1989; Tomita et al., 1999). While these emotional and cognitive forces often operate in tandem, they can also compete, particularly in emotionally complex contexts, raising questions about which type of salience dominates during offline memory consolidation.

Although early research conceptualized sleep as a global stabilizer of newly encoded material (e.g., Stickgold, 2005; Diekelmann and Born, 2010; Ellenbogen et al., 2006; Plihal and Born, 1997), accumulating evidence suggests that sleep selectively consolidates memories tagged as motivationally or cognitively relevant (Stickgold and Walker, 2013; Klinzing et al., 2019; Kim and Payne, 2020). Rather than uniformly strengthening all encoded material, sleep appears to favor memories that align with specific motivational or cognitive cues present during encoding—such as rewards value (e.g., Sterpenich et al., 2021; Fischer and Born, 2009; Asfestani et al., 2020), future utility (e.g., Wilhelm et al., 2011; van Dongen et al., 2012), or emotional significance (e.g., Payne et al., 2012; Payne et al., 2008; Cunningham et al., 2022; Baran et al., 2012).

Specific features of sleep architecture reflect this selectivity: sleep spindles have been linked to enhanced consolidation of both goal-directed (Schabus et al., 2004; Alger et al., 2019; Saletin et al., 2011; Blaskovich et al., 2017) and emotionally salient memories (Kaestner et al., 2013; Rodheim et al., 2023), while REM-associated theta activity has been associated with emotional generalization and associative integration (Nishida et al., 2009; Hutchison and Rathore, 2015; Sopp et al., 2017; Sterpenich et al., 2014; Backus et al., 2016). Together, these findings suggest that sleep does not simply preserve memory—it actively participates in the selection and transformation of memory content, often guided by competing salience signals like emotion and instruction. This selectivity may begin at encoding, where salient memories are hypothesized to receive neuromodulatory “tags”—for example, via dopamine or norepinephrine signaling—that enhance their likelihood of being reactivated during sleep (Rasch and Born, 2013; Lisman et al., 2011). Reactivation during NREM sleep, particularly during slow oscillations and sleep spindles, is thought to support hippocampal–neocortical communication, stabilizing selected memories through synaptic consolidation (Klinzing et al., 2019). During REM sleep, theta activity may facilitate the integration of emotionally or associatively linked material, further shaping the qualitative nature of the memory trace (Nishida et al., 2009; Boyce et al., 2016).

Despite growing evidence that sleep facilitates selective memory consolidation, relatively few studies have examined how multiple salience cues, such as emotional content and top-down instruction, interact to influence what is retained after sleep. While emotionally negative information is often prioritized in memory, it remains unclear whether such content can be intentionally suppressed, and whether sleep reinforces or overrides those intentional goals. Previous work has shown that emotionally charged material can be resistant to directed forgetting (Nowicka et al., 2010; Yang et al., 2016; Yang et al., 2012; Tay and Yang, 2017; Payne and Corrigan, 2007), yet other studies suggest that intentional forgetting remains effective, even for negative stimuli in some circumstances (Chalkia et al., 2023; Hall et al., 2021).

Importantly, the role of sleep physiology in mediating the balance between bottom-up emotional salience and top-down instructional control remains underexplored. Nap-based studies suggest that when these cues compete, instructional relevance tends to dominate. For example, sleep spindles have been shown to predict memory for goal-relevant (but not purely emotional) information, and neutral remember-cued items are often better retained than emotionally negative ones when intentionally prioritized (Alger et al., 2019; Bennion et al., 2016). These findings highlight the primacy of top-down goals in shaping what is consolidated during sleep—even in the presence of emotional salience.

However, these effects have only been demonstrated in daytime naps, which differ meaningfully from nocturnal sleep in duration, sleep architecture, and circadian influences (Bódizs et al., 2022; Whitehurst et al., 2018; Tarokh et al., 2021). Specifically, nocturnal sleep includes a more complete cycling through NREM and REM stages and greater opportunity for deep slow-wave sleep (SWS) and REM theta activity, both of which may uniquely contribute to emotional and goal-related consolidation (Payne et al., 2012; Nishida et al., 2009; Schabus et al., 2004; Saletin et al., 2011). Studying these processes across a full night of sleep provides a more ecologically valid test of how sleep physiology supports or constrains selective memory processing when multiple salience cues are present.

The present study examined how emotional valence, intentional memory instruction (remember vs. forget), and nocturnal sleep physiology interact to influence memory consolidation across a 12 h delay. Participants completed a directed forgetting paradigm involving neutral and negatively valenced words, followed by both immediate recognition and delayed free recall. We conducted two studies: an initial online experiment in which behavioral data were collected remotely, and a second, in-lab replication (Study 2). In Study 2, we additionally recorded sleep architecture using a Sleep Profiler^®^ EEG headband (Advanced Brain Monitoring, Carlsbad, CA) to assess physiological correlates of memory performance. We hypothesized that instructional cues would exert a stronger influence than emotional valence on memory outcomes, and that participants who slept overnight would exhibit a behavioral memory advantage relative to those who remained awake. Moreover, we predicted that specific features of sleep physiology—particularly spindle density and REM theta activity—would be associated with selective consolidation and integration of instructionally and emotionally relevant content.

## 2 Materials and methods – Study 1 (online study)

### 2.1 Participants

Participants were 45 college-aged students. Of these individuals, 22 were in the sleep group, and 23 were in the wake group. Participants were recruited through the college subject pool (Sona Systems^1^) for an online research study. Participants self-selected into the wake or the sleep group.

### 2.2 Task

**FIGURE 1:**
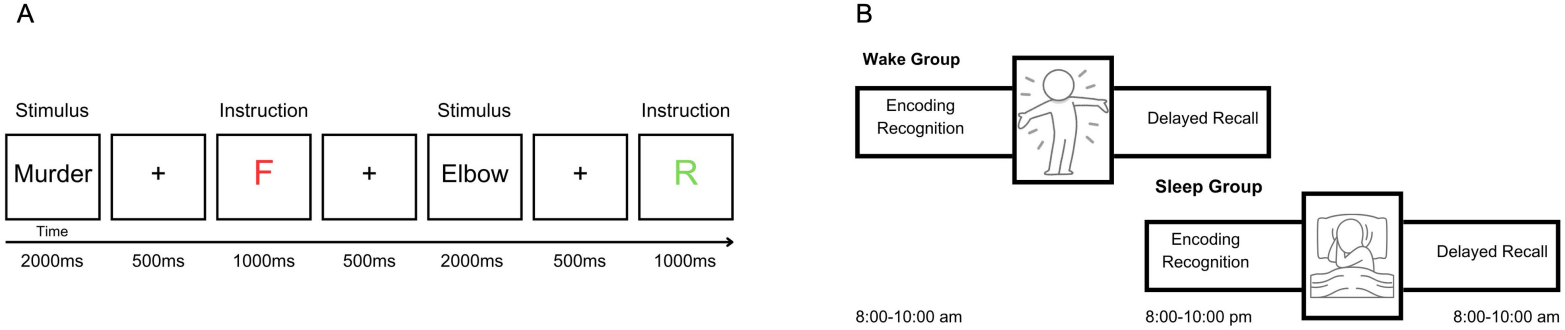
Overview of the directed forgetting paradigm used in both studies. **(A)** Each trial began with a 500 ms fixation cross followed by a word presented for 2,000 ms. A memory cue (“R” for remember or “F” for forget) was displayed for 1,000 ms following each word. **(B)** Participants in the sleep or wake condition completed the task in two sessions separated by a 12 h delay. Session 1 included encoding and an immediate recognition task, and Session 2 included delayed free recall.

The task was a modified version of the directed forgetting paradigm employed by Saletin et al. (2011); Figure 1A). During the encoding phase, participants were shown 100 words on a screen, presented one at a time. Each word was preceded by a 500 ms fixation cross and remained on screen for 2,000 ms. Immediately following each word, an instructional cue was presented for 1,000 ms: a green “R” indicated that the word should be remembered, while a red “F” signaled that the word should be forgotten. Half of the stimuli (*n* = 50) were negatively valenced and half (*n* = 50) were neutral, with all words selected from the Affective Norms for English Words (ANEW; Bradley and Lang, 1999). Words were semi-randomly assigned to the remember or forget conditions, ensuring no significant differences in valence or arousal between instruction conditions within each valence category (see Table 1).

**TABLE 1:** Valence and arousal ratings for negative and neutral words across “remember” and “forget” designations for both Study 1 (online) and Study 2 (in-lab).

Rating type	Valence	Word type	*N*	Mean	SD	t(48)	*P*-value
Valence rating	Negative	Remember	25	1.86	0.168	−1.023	0.312
	Negative	Forget	25	1.91	0.198	–	–
	Neutral	Remember	25	5.14	0.114	−1.216	0.23
	Neutral	Forget	25	5.17	0.092	–	–
Arousal rating	Negative	Remember	25	5.96	1.034	0.091	0.928
	Negative	Forget	25	5.93	0.863	–	–
	Neutral	Remember	25	3.99	0.612	1.01	0.317
	Neutral	Forget	25	3.8	0.711	–	–

Following the encoding phase, participants completed an immediate recognition task. In this task, they were presented with 100 words: 25 previously studied “Remember” words, 25 “Forget” words, and 50 novel foils (25 neutral, 25 negative). For each word, participants indicated whether it was “recognized” or “new.” Upon completion of the recognition task, the first session concluded. After a 12 h delay, participants completed session two, during which they were asked to freely recall any words they remembered from the original encoding list.

### 2.3 Questionnaires

Participants completed several standardized self-report measures. The Positive and Negative Affect Schedule (PANAS; Watson et al., 1988) was administered at both sessions to assess participants’ momentary mood state, with separate subscales for positive and negative affect. The Morningness–Eveningness Questionnaire (MEQ; Horne and Ostberg, 1976) was used to assess chronotype, or individual differences in circadian preference. To assess general sleep quality, participants completed the Pittsburgh Sleep Quality Index (PSQI; Buysse et al., 1989) at the end of the study. All measures yield numerical scores based on participant responses using Likert-type scales. These measures provided information on mood, sleep patterns, and circadian alignment to contextualize individual differences in memory performance.

### 2.4 Procedures

All procedures were approved by the Merrimack College Institutional Review Board. Participants were tested online. Participants were emailed instructions at their designated time (between 8 and 10:00 a.m., or between 8 and 10:00 p.m.; Figure 1B). Within the instructions was a link to a website for this study. Following informed consent, participants were given instructions on the task, and were shown a video of the stimuli and the remember/forget designation. Participants then completed immediate recognition, and also completed the Positive and Negative Affect Scale (PANAS; Watson et al., 1988) before they could sign off for the session. Following a 12 h delay, participants were sent a second website via email at their designated time. In this session, they were asked to freely recall any of the words they saw on the original list. After they could no longer recall any additional words, they were asked to complete the PANAS again, the Morningness and Eveningness Questionnaire (MEQ; Horne and Ostberg, 1976), and the Pittsburgh Sleep Quality Index (PSQI; Buysse et al., 1989).

### 2.5 Free recall scoring

Free recall responses were manually reviewed and scored for accuracy. Minor spelling errors were corrected when the intended word was clearly identifiable (e.g., “muder” for “murder”). Morphological variants of target words were accepted if the root meaning was preserved, such as plural or tense changes (e.g., “doctor” vs. “doctors,” “murder” vs. “murdered”). Only words that matched items from the original encoding list, after accounting for these adjustments, were counted as correct recalls. Words that matched items from the recognition foils list were counted as foil intrusions. Responses that could not be confidently matched to a target word were coded as errors.

### 2.6 Statistical analyses

All analyses were conducted using SPSS version 29.0.1.0. For the immediate recognition task, a 2 (Valence: negative, neutral) × 2 (Instruction: remember, forget) × 2 (Condition: sleep, wake) repeated-measures ANOVA was performed on recognition accuracy, with valence and instruction as within-subject factors and condition as a between-subjects factor. For the delayed free recall task, a similar 2 × 2 × 2 repeated-measures ANOVA was used to analyze the number of correctly recalled words. Separate repeated-measures ANOVAs were conducted on foil intrusions (i.e., words from the recognition task but not from encoding) and error words (i.e., words not shown at any stage), with valence and condition as factors. An alpha level of *p* < 0.05 was used.

## 3 Results – Study 1 (online study)

### 3.1 Questionnaires

There were no significant differences between sleep and wake groups on measures of sleep quality [PSQI: t(28) = −0.483, *p* = 0.633], or chronotype [MEQ: t(27) = 1.376, *p* = 0.180]. There were no differences between groups at either session for the positive or negative affective scores on the PANAS (all *p*’s > 0.465).

### 3.2 Immediate recognition

**FIGURE 2:**
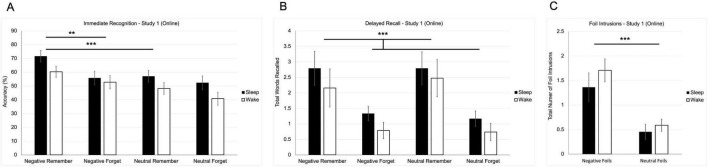
Behavioral results from study 1 (online study). **(A)** Mean recognition accuracy for negative and neutral words across remember and forget instructions. **(B)** Mean number of correctly recalled words as a function of valence and instruction. **(C)** Mean number of foil intrusions (words presented only at recognition but recalled later) by valence. Error bars represent ± 1 SEM. (**p < 0.01; ***p < 0.001).

A two-way repeated-measures ANOVA revealed a significant main effect of instruction [*F*(1, 43) = 11.487, *p* = 0.002; Figure 2A], with remember-cued words recognized more accurately (M = 59.3, SE = 2.5) than forget-cued words (M = 50.4, SE = 2.9). There was also a significant main effect of valence [*F*(1, 43) = 13.109, *p* < 0.001], with negative words (M = 60.1, SE = 2.7) recognized more accurately than neutral words (M = 49.5, SE = 2.8).

A trend toward a main effect of group was observed [*F*(1, 43) = 3.375, *p* = 0.073], with the sleep group (M = 59.2, SE = 3.4) scoring slightly higher than the wake group (M = 50.5, SE = 3.3), suggesting potential baseline differences in recognition performance. However, no significant interactions emerged between valence, instruction, or condition. These findings indicate that recognition performance was driven largely by both top-down cues and emotional salience.

### 3.3 Delayed recall

A two-way repeated-measures ANOVA revealed a significant main effect of instruction [*F*(1, 41) = 20.366, *p* < 0.001; Figure 2B], such that remember-cued words (M = 2.554, SE = 0.379) were recalled more frequently than forget-cued words (M = 1.007, SE = 0.146). There was no significant main effect of valence [*F*(1, 41) = 0.015, *p* = 0.903; Figure 2B]. No significant interactions were observed, and there was no main effect of group on recall performance [*F*(1, 41) = 1.092, *p* = 0.302; Figure 2B], indicating that the sleep condition did not influence total recall.

A repeated measures ANOVA indicated that there was a significant effect of valence on the number of foil intrusions – words that were shown as foils in immediate recognition, but not part of the initial encoding [*F*(1, 26) = 28.908, *p* < 0.001; Figure 2C]. Negative foils (M = 1.535, SE = 0.186) were better recalled than neutral foils (M = 0.521, SE = 0.099), suggesting a memory bias toward emotionally salient content, even when that content was not originally learned. However, there was no effect of condition on recall of foil intrusions [*F*(1, 26) = 0.306, *p* = 0.585] indicating that this emotional memory distortion was not modulated by sleep opportunity. Finally, there was no effect of condition on the total number of errors – words that were never shown in encoding, nor in immediate recognition [*t*(23) = 1.078, *p* = 0.292], suggesting that sleep did not significantly influence overall memory accuracy or false recall of completely novel items.

## 4 Materials and methods – Study 2 (in-lab replication study)

### 4.1 Participants

Participants were 53 young adults (23 sleep, 30 wake; 46 female; average age = 22.26 years). Of the sleep group, 15 participants had useable sleep physiology data. Participants were recruited through the college subject pool (Sona Systems^1^).

### 4.2 Task

The task procedure was identical to that used in Study 1, with the primary difference being the testing environment. Instead of completing the task online via a web platform, participants in Study 2 completed the task in the laboratory using PsychoPy^®^ software (Peirce et al., 2019).

### 4.3 Questionnaires

Participants completed the same standardized self-report measures as in Study 1 (PANAS, MEQ, and PSQI). Participants also reported their age and gender.

### 4.4 Procedures

All procedures for Study 2 were approved by the Merrimack College Institutional Review Board. Study 2 followed the same protocol as Study 1, with the exception that participants were tested in person rather than online. Participants arrived at the laboratory at their assigned time (between 8:00–10:00 a.m. and 8:00–10:00 p.m.) and completed the same task using PsychoPy^®^ software (Peirce et al., 2019). All task instructions, stimuli, and timing remained identical to Study 1. Free recall responses were scored in the same way as Study 1.

Participants in the sleep condition also wore a Sleep Profiler^®^ EEG headband (Advanced Brain Monitoring, Carlsbad, CA) overnight to record sleep architecture. The following morning, they returned to complete the delayed recall task. As in Study 1, participants completed the PANAS (Watson et al., 1988) before and after the 12 h delay, and also completed the MEQ (Horne and Ostberg, 1976) and PSQI (Buysse et al., 1989) at the final session.

### 4.5 Sleep physiology collection

In Study 2, sleep architecture was recorded using the Sleep Profiler^®^ EEG headband system (Advanced Brain Monitoring, Carlsbad, CA), a validated ambulatory device that captures frontal EEG, EOG, and EMG signals. The device includes three gold-plated electrodes embedded in the headband to record differential EEG from two frontopolar derivations (AF7–AF8) referenced to Fpz, in accordance with the international 10–20 system. EEG signals were sampled at 256 Hz, with a 0.1 Hz high pass, and 80 Hz low pass filter applied. The system also includes an embedded accelerometer and capacitive sensors to monitor body position, snoring, and respiratory patterns. Participants in the sleep condition wore the headband overnight in their home environments. Data were scored automatically using the manufacturer’s proprietary algorithms, which have been validated against registered polysomnographic technologists (Levendowski et al., 2017), and manually reviewed for accuracy using Sleep Profiler Analysis Software. Standard AASM criteria were applied to quantify time spent in each sleep stage (NREM1, NREM2, NREM3, and REM), as well as spectral power in relevant frequency bands. Specifically, spindle count, delta power (0.5–4 Hz) during NREM2 and NREM3, and REM theta power (4–8 Hz) were extracted for correlational analyses with memory performance.

Of the 23 participants in the sleep group, three participants chose not to wear the headband, one took off the headband in the middle of the night, two did not turn on the headband to record data, and two participant’s data had uploading errors. A total of 15 participants therefore had useable PSG data for analysis.

### 4.6 Statistical analyses

The same analytic structure was used for Study 2 as in Study 1. Recognition and recall data were analyzed using 2 (Valence: negative, neutral) × 2 (Instruction: remember, forget) × 2 (Condition: sleep, wake) repeated-measures ANOVAs, with follow-up *t*-tests used to interpret significant main effects and interactions. All pairwise comparisons were Bonferroni-corrected where applicable. Separate ANOVAs examined foil intrusions and error words by valence and condition. In addition to behavioral analyses, Pearson’s correlations were conducted within the sleep group between physiological sleep variables (i.e., spindle count, NREM3 percentage, delta power during NREM2/3, and REM theta power) and memory outcomes (e.g., recall of negative remember-cued words, total recall, and negative foil intrusions).

## 5 Results – Study 2 (in-lab replication study)

### 5.1 Questionnaires

There were no significant differences between sleep and wake groups on measures of sleep quality [PSQI: t(23) = −0.486, *p* = 0.631], or chronotype [MEQ: t(23) = −0.845, *p* = 0.407]. There were no differences between groups at either session for the positive or negative affective scores on the PANAS (all *p*’s > 0.186).

### 5.2 Immediate recognition

**FIGURE 3:**
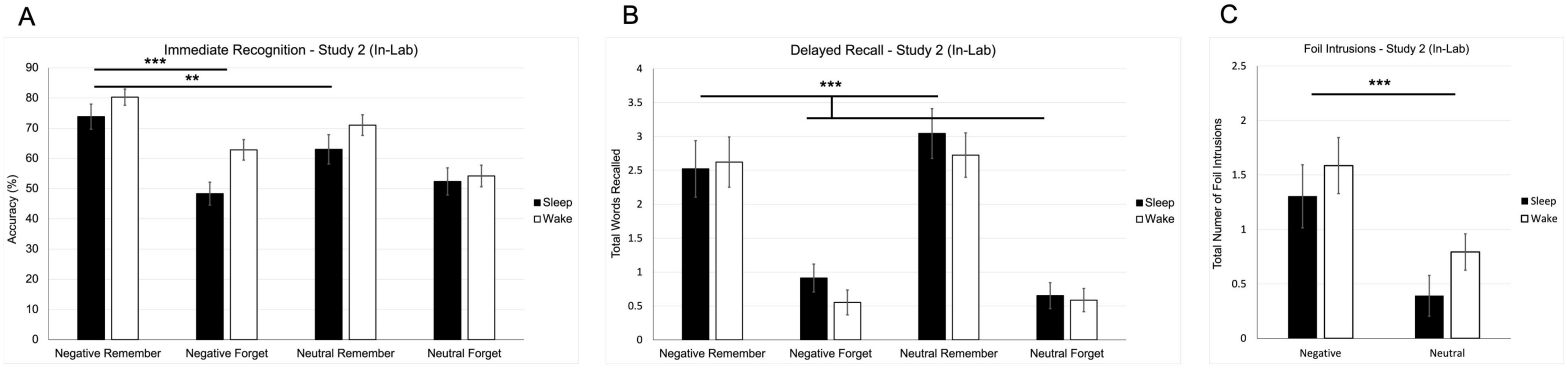
Behavioral results from Study 2 (in-lab replication study). **(A)** Mean recognition accuracy for negative and neutral words across remember and forget instructions. **(B)** Mean number of correctly recalled words as a function of valence and instruction. **(C)** Mean number of foil intrusions (words presented only at recognition but recalled later) by valence. Error bars represent ± 1 SEM. (**p < 0.01; ***p < 0.001).

A two-way repeated-measures ANOVA revealed significant main effects of valence [*F*(1, 49) = 7.281, *p* = 0.01] and instruction [*F*(1, 49) = 67.186, *p* < 0.001] on recognition accuracy, indicating that both emotional content and top-down cues influenced recognition performance (Figure 3A). There was also a significant valence × instruction interaction [*F*(1, 49) = 4.962, *p* = 0.031; Figure 3B], suggesting that emotion enhanced memory selectively when paired with a goal-directed cue to remember. To interpret this interaction, we conducted three pairwise comparisons between relevant word types. Bonferroni correction was applied to control for multiple comparisons (α = 0.05÷3 = 0.017). Recognition accuracy was significantly higher for negative remember words than for negative forget words [*t*(50) = 9.291, *p* < 0.001] and neutral remember words [*t*(50) = 3.423, *p* = 0.001]; both comparisons remained significant under the corrected alpha. There was no significant difference between negative forget and neutral forget words [*t*(50) = 1.202, *p* = 0.235; Figure 3A]. These findings suggest that recognition was primarily shaped by intentional encoding, with emotion amplifying memory only when aligned with top-down goals.

### 5.3 Delayed recall

A two-way repeated-measures ANOVA on delayed free recall revealed a significant main effect of instruction [*F*(1, 50) = 65.773, *p* < 0.001; Figure 3B], indicating that remember-cued words were recalled more accurately than forget-cued words. There were no significant main effects of valence or condition (sleep vs. wake) on correct recall, demonstrating that instructional relevance, rather than emotional content or sleep group, primarily guided memory performance at delayed recall.

To examine false memory, a separate repeated-measures ANOVA was conducted on foil intrusions—words recalled from the recognition phase but not originally encoded. This analysis revealed a significant main effect of valence [*F*(1, 50) = 14.081, *p* < 0.001; Figure 3C], with more negative foils (M = 1.445, SE = 0.193) recalled than neutral foils (M = 0.592, SE = 0.125), suggesting that emotional salience increased vulnerability to memory distortion. No significant main effect of condition [*F*(1, 50) = 2.146, *p* = 0.149] or valence × condition interaction [*F*(1, 50) = 0.07, *p* = 0.793] was observed.

### 5.4 Sleep-memory associations

**FIGURE 4:**
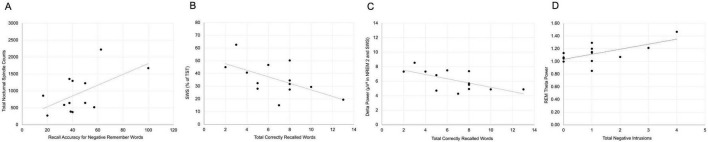
Sleep–memory associations in Study 2. **(A)** Sleep spindle count positively correlated with recall accuracy for negative remember-cued words. **(B)** Total recall negatively correlated with percentage of time spent in Slow Wave Sleep (SWS). **(C)** Delta power during NREM2 and SWS was negatively correlated with total recall. **(D)** REM theta power positively correlated with negative foil intrusions.

Within the sleep group, recall accuracy for negative remember-cued words was positively correlated with sleep spindle count (*r* = 0.150, *p* = 0.041; Figure 4A), supporting the possible role of spindles in the targeted consolidation of emotionally salient, goal-relevant information. In contrast, total recall was negatively associated with both the percentage of the night spent in SWS (*r* = −0.584, *p* = 0.036; Figure 4B) and delta power during NREM2 and SWS (*r* = −0.631, *p* = 0.021; Figure 4C), suggesting a potential trade-off wherein deeper slow-wave activity may interfere with broader memory accessibility.

Additionally, negative foil intrusions were positively correlated with REM theta power (*r* = 0.635, *p* = 0.020; Figure 4D), consistent with the hypothesis that REM-related oscillatory activity contributes to emotional memory generalization or misattribution. These findings highlight that, even in the absence of a behavioral benefit of sleep, distinct sleep physiological markers tracked selective memory enhancement and emotional distortion.

### 5.5 Discussion

Across two studies, we investigated how emotional salience and top-down instruction interact to influence memory consolidation following a 12 h delay that included either wakefulness or sleep. While behavioral measures did not reveal a sleep-related advantage in memory performance, our findings support several key conclusions: (1) instruction to remember strongly enhanced both recognition and recall relative to instruction to forget; (2) emotional salience increased the likelihood of false recall, particularly for foils introduced during immediate recognition; and (3) physiological markers of sleep, especially spindle activity and REM theta, predicted selective consolidation and emotional memory distortion in the absence of a main behavioral effect of sleep.

Consistent with prior work on directed forgetting (e.g., Anderson and Green, 2001; Bjork, 1989), both studies demonstrated robust effects of top-down instruction on memory: remember-cued words were recalled and recognized more accurately than forget-cued words. These findings align with recent evidence that intentional encoding goals exert a dominant influence on memory consolidation, even when emotionally salient information is present (Alger et al., 2019; Bennion et al., 2016). Emotional valence also enhanced recognition, but this effect was largely dependent on whether the item was goal-relevant (e.g., negative remember words). In contrast, emotion alone did not enhance delayed recall, nor did it override intentional forgetting instructions—suggesting that top-down control reliably constrains memory, even for negative content. This pattern—where emotion enhanced recognition but not recall—is consistent with dual-process theories of memory (Yonelinas, 2002), and with evidence that emotion often disproportionately supports familiarity-based recognition rather than effortful recollection (Sharot and Yonelinas, 2008).

Notably, emotionally negative foils were more likely to be falsely recalled than neutral foils, replicating prior findings that emotionally salient but non-encoded content may be more susceptible to intrusions (Brainerd et al., 2010; Lee and Fernandes, 2018). This pattern was consistent across both studies and did not differ by sleep condition, indicating that emotion-driven memory distortion may be relatively robust to sleep–wake state. Importantly, the replication of behavioral findings across both studies—despite differences in testing modality (online vs. in-lab)—adds confidence to the robustness of our results. However, only participants in the in-lab study (Study 2) provided EEG data. The addition of EEG recording in Study 2 allowed us to build on these replicated behavioral effects with novel physiological insights.

Although behavioral memory did not differ by sleep condition, several features of sleep architecture were significantly associated with selective recall. Sleep spindle count was positively correlated with memory for negative remember words, echoing prior research linking spindles to targeted memory consolidation (Saletin et al., 2011; Schabus et al., 2004). Conversely, time spent in SWS and associated delta power were negatively correlated with total recall, suggesting a potential trade-off between deep slow-wave activity and memory accessibility; this finding is supported by previous research that suggests that SWS and delta waves in particular may support forgetting (Poe, 2017; Kim et al., 2019). REM theta activity was positively associated with negative foil intrusions, consistent with the view that REM contributes to emotional memory integration or generalization (Nishida et al., 2009; Hutchison and Rathore, 2015; Sopp et al., 2017; Sterpenich et al., 2014; Backus et al., 2016).

Together, these findings support the view that memory consolidation during sleep is not uniform, but selective, shaped by both motivational goals and emotional salience. Crucially, it was sleep physiology, rather than the mere presence of sleep, that tracked selective memory outcomes, highlighting the importance of looking beyond binary sleep–wake comparisons to uncover the mechanisms guiding memory retention and distortion. The absence of a main behavioral sleep effect despite strong physiological predictors of memory consolidation, aligns with growing evidence that group-level sleep vs. wake comparisons may underestimate more nuanced sleep-related effects. Several factors may contribute to this dissociation. First, individual variability in sleep architecture—including differences in spindle density, REM theta activity, and time spent in SWS—can mask group-level effects, especially when sample sizes for EEG are modest. Second, wake participants may engage in rehearsal or ruminative processing during the 12 h delay, particularly for emotionally salient content, which could dampen group differences. Finally, it is possible that sleep’s influence is more targeted than global, enhancing memory for specific types of content (e.g., goal-relevant or emotionally incongruent items) rather than producing a uniform behavioral advantage. This interpretation is supported by our findings that sleep physiology—but not sleep condition—was associated with selective recall and memory distortion.

Importantly, this study is one of the first to extend nap-based findings to overnight sleep in a memory task involving competing salience cues, offering a more comprehensive view of how selective consolidation unfolds across the full sleep cycle. Whereas nap-based research has shown that spindles support goal-relevant recall (e.g., Alger et al., 2019) and that top-down cues may outweigh emotional salience (Bennion et al., 2016), our results demonstrate that similar mechanisms are active across a full night of sleep. The presence of extended SWS and REM theta activity may allow for both deeper downscaling and greater associative integration, processes that are often less accessible in the more fragmented architecture of a nap. This distinction underscores the value of using nocturnal sleep paradigms to test theories of emotional and intentional memory consolidation. Lastly, by replicating behavioral findings across distinct testing contexts and extending them with physiological measures, this study provides a robust and ecologically valid demonstration of selective memory consolidation mechanisms.

While this study helps clarify how emotional and cognitive cues shape memory consolidation, several limitations should be noted. First, the sample size for the sleep EEG group was modest, limiting statistical power for sleep–memory associations. Second, the sample was largely college-students, limiting generalizability. Third, in the online study we had no measures of overnight sleep time or quality on the experimental night, although PSQI results suggest no group differences in typical sleep quality. Fourth, although the 12 h AM/PM testing schedule is widely used in sleep–wake memory designs, we cannot fully rule out the influence of circadian factors on encoding or recall performance. Finally, future studies might examine whether individual differences in affective traits or sleep quality moderate these selective consolidation effects.

In sum, these findings contribute to growing evidence that memory is not passively stabilized during sleep, but actively shaped by a combination of what we value, what we intend to retain, and how our brains process this information offline. Instructional relevance emerged as a stronger driver of memory than emotion, and sleep architecture provided a critical window into the mechanisms by which selectivity is implemented in the absence of a clear behavioral sleep effect. These results underscore the idea that top-down control may play a more decisive role than bottom-up emotional salience in determining which memories are consolidated, particularly when both cues are in competition.

## Data Availability

The datasets generated for this study can be found in the Open Science Framework repository: https://osf.io/egtu2/.

## Footnotes

1
https://www.sona-systems.com/

## References

[B1] Adelman J. S. Estes Z. (2013). Emotion and memory: A recognition advantage for positive and negative words independent of arousal. *Cognition* 129 530–535. 10.1016/j.cognition.2013.08.014 24041838

[B2] Alger S. E. Chen S. Payne J. D. (2019). Do different salience cues compete for dominance in memory over a daytime nap?”. *Neurobiol. Learn. Mem.* 160 48–57. 10.1016/j.nlm.2018.06.005 29906574 PMC6291383

[B3] Anderson M. C. Green C. (2001). Suppressing unwanted memories by executive control. *Nature* 410 366–369. 10.1038/35066572 11268212

[B4] Anderson M. C. Hulbert J. C. (2021). Active forgetting: Adaptation of memory by prefrontal control. *Annu. Rev. Psychol.* 72 1–36. 10.1146/annurev-psych-072720-094140 32928060

[B5] Asfestani M. Brechtmann V. Santiago J. Peter A. Born J. Feld G. (2020). Consolidation of reward memory during sleep does not require dopaminergic activation. *J. Cogn. Neurosci.* 32 1688–1703. 10.1162/jocn_a_01585 32459129

[B6] Backus A. R. Schoffelen J.-M. Szebényi S. Hanslmayr S. Doeller C. F. (2016). Hippocampal-prefrontal theta oscillations support memory integration. *Curr. Biol.* 26 450–457. 10.1016/j.cub.2015.12.048 26832442

[B7] Baran B. Pace-Schott E. F. Ericson C. Spencer R. M. C. (2012). Processing of emotional reactivity and emotional memory over sleep. *J. Neurosci.* 32 1035–1042. 10.1523/JNEUROSCI.2532-11.2012 22262901 PMC3548452

[B8] Bennion K. A. Payne J. D. Kensinger E. A. (2016). The impact of napping on memory for future-relevant stimuli: Prioritization among multiple salience cues. *Behav. Neurosci.* 130 281–289. 10.1037/bne0000142 27214500

[B9] Bjork R. (1989). *Retrieval Inhibition as an Adaptive Mechanism in Human Memory.* Los Angeles, CA: University of California

[B10] Blaskovich B. Szőllősi Á Gombos F. Racsmány M. Simor P. (2017). The benefit of directed forgetting persists after a daytime nap: The role of spindles and rapid eye movement sleep in the consolidation of relevant memories. *Sleep* 40:zsw076. 10.1093/sleep/zsw076 28364418

[B11] Bódizs R. Horváth C. Szalárdy O. Ujma P. Simor P. Gombos F. (2022). Sleep-spindle frequency: Overnight dynamics, afternoon nap effects, and possible circadian modulation. *J. Sleep Res.* 31:e13514. 10.1111/jsr.13514 34761463

[B12] Boyce R. Glasgow S. D. Williams S. Adamantidis A. (2016). Causal evidence for the role of REM sleep theta rhythm in contextual memory consolidation. *Science* 352 812–816. 10.1126/science.aad5252 27174984

[B13] Bradley M. M. Lang P. J. (1999). *Affective Norms for English Words (ANEW): Instruction Manual and Affective Ratings.* Gainesville, FL: University of Florida.

[B14] Brainerd C. J. Holliday R. E. Reyna V. F. Yang Y. Toglia M. P. (2010). Developmental reversals in false memory: Effects of emotional valence and arousal. *J. Exp. Child Psychol.* 107 137–154. 10.1016/j.jecp.2010.04.013 20547393 PMC2904859

[B15] Buysse D. Reynolds C. III Monk T. Berman S. Kupfer D. (1989). The pittsburgh sleep quality index: A new instrument for psychiatric practice and research. *Psychiatry Res.* 28 193–213. 10.1016/0165-1781(89)90047-4 2748771

[B16] Chalkia A. Vanhasbroeck N. Van Oudenhove L. Kindt M. Beckers T. (2023). Emotional associative memory is disrupted by directed forgetting. *Commun. Psychol.* 1 1–12. 10.1038/s44271-023-00024-x 39242722 PMC11332221

[B17] Cunningham T. J. Stickgold R. Kensinger E. A. (2022). Investigating the effects of sleep and sleep loss on the different stages of episodic emotional memory: A narrative review and guide to the future. *Front. Behav. Neurosci.* 16:910317. 10.3389/fnbeh.2022.910317 36105652 PMC9466000

[B18] Diekelmann S. Born J. (2010). The memory function of sleep. *Nat. Rev. Neurosci.* 11 114–126. 10.1038/nrn2762 20046194

[B19] Ellenbogen J. M. Hulbert J. C. Stickgold R. Dinges D. F. Thompson-Schill S. L. (2006). Interfering with theories of sleep and memory: Sleep, declarative memory, and associative interference. *Curr. Biol.* 16 1290–1294. 10.1016/j.cub.2006.05.024 16824917

[B20] Fiacconi C. M. Dekraker J. Köhler S. (2015). Psychophysiological evidence for the role of emotion in adaptive memory. *J. Exp. Psychol.* 144 925–933. 10.1037/xge0000097 26280160

[B21] Fischer S. Born J. (2009). Anticipated reward enhances offline learning during sleep. *J. Exp. Psychol.* 35 1586–1593. 10.1037/a0017256 19857029

[B22] Hall K. Fawcett E. Hourihan K. Fawcett J. (2021). Emotional memories are (usually) harder to forget: A meta-analysis of the item-method directed forgetting literature. *Psychon. Bull. Rev.* 28 1313–1326. 10.3758/s13423-021-01914-z 33846935

[B23] Horne J. A. Ostberg O. (1976). A self-assessment questionnaire to determine morningness-eveningness in human circadian rhythms. *Int. J. Chronobiol.* 4 97–110. 1027738

[B24] Hutchison I. C. Rathore S. (2015). The role of REM sleep theta activity in emotional memory. *Front. Psychol.* 6:1439. 10.3389/fpsyg.2015.01439 26483709 PMC4589642

[B25] Jenkins J. G. Dallenbach K. M. (1924). Obliviscence during sleep and waking. *Am. J. Psychol.* 35 605–612. 10.2307/1414040

[B26] Joensen B. Harrington M. Berens S. Cairney S. Gaskell M. Horner A. (2022). Targeted memory reactivation during sleep can induce forgetting of overlapping memories. *Learn. Mem.* 29 401–411. 10.1101/lm.053594.122 36253007 PMC9578373

[B27] Kaestner E. J. Wixted J. T. Mednick S. C. (2013). Pharmacologically increasing sleep spindles enhances recognition for negative and high-arousal memories. *J. Cogn. Neurosci.* 25 1597–1610. 10.1162/jocn_a_00433 23767926

[B28] Kensinger E. A. (2004). Remembering Emotional Experiences: The Contribution of Valence and Arousal. *Rev. Neurosci.* 15 241–251. 10.1515/revneuro.2004.15.4.241 15526549

[B29] Kensinger E. A. (2009). Remembering the details: Effects of emotion. *Emot. Rev.* 1 99–113. 10.1177/1754073908100432 19421427 PMC2676782

[B30] Kensinger E. A. Schacter D. L. (2008). *Memory and Emotion: Handbook of Emotions*, 3rd Edn. New York, NY: The Guilford Press.

[B31] Kim J. Gulati T. Ganguly K. (2019). Competing roles of slow oscillations and delta waves in memory consolidation versus forgetting. *Cell* 179 514–526.e13. 10.1016/j.cell.2019.08.040 31585085 PMC6779327

[B32] Kim S. Y. Payne J. D. (2020). Neural correlates of sleep, stress, and selective memory consolidation. *Curr. Opin. Behav.* 33 57–64. 10.1016/j.cobeha.2019.12.009

[B33] Klinzing J. G. Niethard N. Born J. (2019). Mechanisms of systems memory consolidation during sleep. *Nat. Neurosci.* 22 1598–1610. 10.1038/s41593-019-0467-3 31451802

[B34] Lee C. Fernandes M. A. (2018). Emotional encoding context leads to memory bias in individuals with high anxiety. *Brain Sci.* 8:1. 10.3390/brainsci8010006 29280957 PMC5789337

[B35] Lerner I. Lupkin S. Tsai A. Khawaja A. Gluck M. (2021). Sleep to remember, sleep to forget: Rapid eye movement sleep can have inverse effects on recall and generalization of fear memories. *Neurobiol. Learn. Mem.* 180:107413. 10.1016/j.nlm.2021.107413 33609741

[B36] Levendowski D. Ferini-Strambi L. Gamaldo C. Cetel M. Rosenberg R. Westbrook P. (2017). The accuracy, night-to-night variability, and stability of frontopolar sleep electroencephalography biomarkers. *J. Clin. Sleep Med.* 13 791–803. 10.5664/jcsm.6618 28454598 PMC5443740

[B37] Lisman J. Grace A. Duzel E. (2011). A neoHebbian framework for episodic memory; role of dopamine-dependent late LTP. *Trends Neurosci.* 34 536–547. 10.1016/j.tins.2011.07.006 21851992 PMC3183413

[B38] MacDonald K. J. Cote K. A. (2021). Contributions of post-learning REM and NREM sleep to memory retrieval. *Sleep Med. Rev.* 59:101453. 10.1016/j.smrv.2021.101453 33588273

[B39] Mather M. Sutherland M. R. (2011). Arousal-biased competition in perception and memory. *Perspect. Psychol. Sci.* 6 114–133. 10.1177/1745691611400234 21660127 PMC3110019

[B40] McGaugh J. L. (2004). The amygdala modulates the consolidation of memories of emotionally arousing experiences. *Annu. Rev. Neurosci.* 27 1–28. 10.1146/annurev.neuro.27.070203.144157 15217324

[B41] Nishida M. Pearsall J. Buckner R. Walker M. (2009). REM sleep, prefrontal theta, and the consolidation of human emotional memory. *Cereb. Cortex* 19 1158–1166. 10.1093/cercor/bhn155 18832332 PMC2665156

[B42] Nowicka A. Marchewka A. Jednoróg K. Tacikowski P. Brechmann A. (2010). Forgetting of emotional information is hard: An fMRI study of directed forgetting. *Cereb. Cortex* 27:bhq117. 10.1093/cercor/bhq117 20584747

[B43] Paller K. A. Creery J. D. Schechtman E. (2021). Memory and sleep: How sleep cognition can change the waking mind for the better. *Annu. Rev. Psychol.* 72 123–150. 10.1146/annurev-psych-010419-050815 32946325 PMC7983127

[B44] Paller K. A. Voss J. L. (2004). Memory reactivation and consolidation during sleep. *Learn. Mem.* 11 664–670. 10.1101/lm.75704 15576883 PMC534694

[B45] Payne B. K. Corrigan E. (2007). Emotional constraints on intentional forgetting. *J. Exp. Soc. Psychol.* 43 780–786. 10.1016/j.jesp.2006.07.005

[B46] Payne J. Chambers A. Kensinger E. (2012). Sleep promotes lasting changes in selective memory for emotional scenes. *Front. Integr. Neurosci.* 6:108. 10.3389/fnint.2012.00108 23181013 PMC3503264

[B47] Payne J. D. Stickgold R. Swanberg K. Kensinger E. A. (2008). Sleep preferentially enhances memory for emotional components of scenes. *Psychol. Sci.* 19 781–788. 10.1111/j.1467-9280.2008.02157.x 18816285 PMC5846336

[B48] Peirce J. Gray J. R. Simpson S. MacAskill M. Höchenberger R. Sogo H. (2019). PsychoPy2: Experiments in behavior made easy. *Behav. Res. Methods*. 51, 195–203. 10.3758/s13428-018-01193-y 30734206 PMC6420413

[B49] Pereira D. R. Teixeira-Santos A. C. Sampaio A. Pinheiro A. P. (2023). Examining the effects of emotional valence and arousal on source memory: A meta-analysis of behavioral evidence. *Emotion* 23 1740–1763. 10.1037/emo0001188 36480404

[B50] Plihal W. Born J. (1997). Effects of early and late nocturnal sleep on declarative and procedural memory. *J. Cogn. Neurosci.* 9 534–547. 10.1162/jocn.1997.9.4.534 23968216

[B51] Poe G. R. (2017). Sleep is for forgetting. Dual perspectives. *J. Neurosci.* 37 464–473. 10.1523/JNEUROSCI.0820-16.2017 28100731 PMC5242402

[B52] Rasch B. Born J. (2013). About sleep’s role in memory. *Physiol. Rev.* 93 681–766. 10.1152/physrev.00032.2012 23589831 PMC3768102

[B53] Rodheim K. Kainec K. Noh E. Jones B. Spencer R. M. C. (2023). Emotional memory consolidation during sleep is associated with slow oscillation–spindle coupling strength in young and older adults. *Learn. Mem.* 30 237–244. 10.1101/lm.053685.122 37770106 PMC10547370

[B54] Rudoy J. D. Voss J. L. Westerberg C. E. Paller K. A. (2009). Strengthening individual memories by reactivating them during sleep. *Science* 326 1079–1079. 10.1126/science.1179013 19965421 PMC2990343

[B55] Ryan T. J. Frankland P. W. (2022). Forgetting as a form of adaptive engram cell plasticity. *Nat. Rev. Neurosci.* 23 173–186. 10.1038/s41583-021-00548-3 35027710

[B56] Saletin J. M. Goldstein A. N. Walker M. P. (2011). The role of sleep in directed forgetting and remembering of human memories. *Cereb. Cortex* 31:bhr034. 10.1093/cercor/bhr034 21459838 PMC3183424

[B57] Schabus M. Gruber G. Parapatics S. Sauter C. Klösch G. Anderer P. (2004). Sleep spindles and their significance for declarative memory consolidation. *Sleep* 27 1479–1485. 10.1093/sleep/27.7.1479 15683137

[B58] Sharot T. Yonelinas A. P. (2008). Differential time-dependent effects of emotion on recollective experience and memory for contextual information. *Cognition* 106 538–547. 10.1016/j.cognition.2007.03.002 17451666

[B59] Sopp M. Michael T. Weeß H. Mecklinger A. (2017). Remembering specific features of emotional events across time: The role of REM sleep and prefrontal theta oscillations. *Cognit. Affect. Behav. Neurosci.* 17 1186–1209. 10.3758/s13415-017-0542-8 29063522

[B60] Sterpenich V. Schmidt C. Albouy G. Matarazzo L. Vanhaudenhuyse A. Boveroux P. (2014). Memory reactivation during rapid eye movement sleep promotes its generalization and integration in cortical stores. *Sleep* 37 1061–1075. 10.5665/sleep.3762 24882901 PMC4015380

[B61] Sterpenich V. van Schie Mojca K. Catsiyannis M. Ramyead A. Perrig S. Yang H. (2021). Reward biases spontaneous neural reactivation during sleep. *Nat. Commun.* 12:4162. 10.1038/s41467-021-24357-5 34230462 PMC8260738

[B62] Stickgold R. (2005). Sleep-dependent memory consolidation. *Nature* 437 1272–1278. 10.1038/nature04286 16251952

[B63] Stickgold R. Walker M. P. (2013). Sleep-dependent memory triage: Evolving generalization through selective processing. *Nat. Neurosci.* 16 139–145. 10.1038/nn.3303 23354387 PMC5826623

[B64] Tarokh L. Van Reen E. Achermann P. Carskadon M. (2021). Naps not as effective as a night of sleep at dissipating sleep pressure. *J. Sleep Res.* 30 e13295. 10.1111/jsr.13295 33622020 PMC10948110

[B65] Tay P. K. C. Yang H. (2017). Angry faces are more resistant to forgetting than are happy faces: Directed forgetting effects on the identity of emotional faces. *J. Cogn. Psychol.* 29 855–865. 10.1080/20445911.2017.1323907

[B66] Tomita H. Ohbayashi M. Nakahara K. Hasegawa I. Miyashita Y. (1999). Top-down signal from prefrontal cortex in executive control of memory retrieval. *Nature* 401 699–703. 10.1038/44372 10537108

[B67] van Dongen E. V. Thielen J. W. Takashima A. Barth M. Fernández G. (2012). Sleep supports selective retention of associative memories based on relevance for future utilization. *PLoS One* 7:e43426. 10.1371/journal.pone.0043426 22916259 PMC3420871

[B68] Walker R. Russo V. (2004). Memory consolidation and forgetting during sleep: A neural network model. *Neural Process. Lett.* 19 147–156. 10.1023/B:NEPL.0000023445.96334.eb

[B69] Watson D. Clark L. A. Tellegen A. (1988). Development and validation of brief measures of positive and negative affect: The PANAS scales. *J. Pers. Soc. Psychol.* 54 1063–1070. 10.1037//0022-3514.54.6.1063 3397865

[B70] Whitehurst L. N. Naji M. Mednick S. C. (2018). Comparing the cardiac autonomic activity profile of daytime naps and nighttime sleep. *Neurobiol. Sleep Circ. Rhythms* 5 52–57. 10.1016/j.nbscr.2018.03.001 31236511 PMC6584676

[B71] Wilhelm I. Diekelmann S. Molzow I. Ayoub A. Mölle M. Born J. (2011). Sleep selectively enhances memory expected to be of future relevance. *J. Neurosci.* 31 1563–1569. 10.1523/JNEUROSCI.3575-10.2011 21289163 PMC6623736

[B72] Yang T. Lei X. Anderson M. (2016). Decreased inhibitory control of negative information in directed forgetting. *Int. J. Psychophysiol.* 100 44–51. 10.1016/j.ijpsycho.2015.09.007 26386395

[B73] Yang W. Liu P. Xiao X. Li X. Zeng C. Qiu J. (2012). Different neural substrates underlying directed forgetting for negative and neutral images: An event-related potential study. *Brain Res.* 1441 53–63. 10.1016/j.brainres.2011.10.042 22285435

[B74] Yonelinas A. P. (2002). The nature of recollection and familiarity: A review of 30 years of research. *J. Mem. Lang.* 46 441–517. 10.1006/jmla.2002.2864

